# EMAST Is Associated with a Poor Prognosis in Microsatellite Instable Metastatic Colorectal Cancer

**DOI:** 10.1371/journal.pone.0124538

**Published:** 2015-04-17

**Authors:** Sabine Venderbosch, Shannon van Lent—van Vliet, Anton F. J. de Haan, Marjolijn J. Ligtenberg, Monique Goossens, Cornelis J. A. Punt, Miriam Koopman, Iris D. Nagtegaal

**Affiliations:** 1 Department of Pathology, Radboud university medical center, PO Box 9101–6500 HB, Nijmegen, The Netherlands; 2 Department for Health Evidence, Radboud university medical center, PO Box 9101–6500 HB, Nijmegen, The Netherlands; 3 Department of Medical Oncology, Academic Medical Center, University of Amsterdam, PO Box 22660–1100 DD, Amsterdam, The Netherlands; 4 Department of Human Genetics, Radboud university medical center, PO Box 9101–6500 HB, Nijmegen, The Netherlands; 5 Department of Medical Oncology, University Medical Center Utrecht, PO Box 85500–3508 GA, Utrecht, The Netherlands; Sapporo Medical University, JAPAN

## Abstract

**Purpose:**

To determine the frequency and prognostic value of elevated microsatellite alterations at selected tetranucleotide repeats (EMAST) in metastatic colorectal cancer (mCRC) patients in relation to microsatellite instability (MSI) status and MSH3 protein expression.

**Material and Methods:**

The frequency of EMAST was evaluated in mCRC patients with MSI tumors and microsatellite stable (MSS) tumors. A literature overview was performed to compare the frequency of EMAST in our study with existing data. Immunohistochemistry for MSH3 was compared with EMAST status. Outcome was studied in terms of overall survival (OS) of mCRC patients with MSI and MSS tumors.

**Results:**

EMAST was evaluated in 89 patients with MSI tumors (including 39 patients with Lynch syndrome) and 94 patients with MSS tumors. EMAST was observed in 45.9% (84 out of 183) of patients, with an increased frequency in MSI tumors (79.8% versus 13.8%, p < 0.001). We found no correlation between EMAST and MSH3 protein expression. There was no effect of EMAST on prognosis in patients with MSS tumors, but patients with MSI / non-EMAST tumors had a significantly better prognosis than patients with MSI / EMAST tumors (OS: HR 3.22, 95% CI 1.25-8.30).

**Conclusion:**

Frequency of EMAST was increased in mCRC patients with MSI tumors, compared to MSS tumors. Our data suggest that the presence of EMAST correlates with worse OS in these patients. There was no effect of EMAST on the prognosis of patients with MSS tumors. A limitation of our study is the small number of patients in our subgroup analysis.

## Introduction

Colorectal cancer (CRC) carcinogenesis is a multistep process in which different pathways are involved, among which microsatellite instability (MSI) is important [1–3]. MSI is characterized by a deficient mismatch repair system, which leads to cancer development through the accumulation of unrepaired frame shift mutations in simple repeat sequences or microsatellites [4]. To date several mismatch repair (MMR) proteins have been identified in humans: MSH2, MSH3, MSH6, MLH1 and PMS2. MSH2 forms a heterodimer with MSH6 or MSH3, giving rise to MutSα or MutSβ, respectively [5]. MutSα recognizes single base-pair mismatches and small insertion-deletion loops (IDLs), whereas MutSβ preferentially recognizes larger mismatches and IDLs. Furthermore, MLH1 and PMS2 form MutLα, which acts as a molecular matchmaker. In addition to the primary MMR defect, secondary loss of MMR proteins can occur as a consequence of *MSH3* and *MSH6* frame shift mutations promoted by *MLH1* inactivation [6,7] or because of MSH3 and MSH6 protein degradation in tumors not expressing their heterodimeric partner MSH2 [8,9]. As a result, single or combined defects of MMR subunits (MutSα, MutSβ and MutL) can variably underlie the genetic instability of MSI tumors. Germline alterations of MMR genes are the cause of MSI in Lynch syndrome patients [10]. MSI is also observed in 10–20% of patients with sporadic CRC, usually due to promoter hypermethylation of the *MLH1* gene [11,12]. MSI tumors have distinctive features, such as location in the proximal colon, a high incidence of lymphocytic infiltrate, a poorly differentiated, mucinous or signet ring histology [13]. MSI tumors are associated with a favorable prognosis in early stage colon cancer [14].

A distinct form of MSI is observed in several types of cancers and is called ‘elevated microsatellite alterations at selected tetranucleotide repeats’ (EMAST) in contrast to mono-, and dinucleotide based instability in common MSI [15–20]. Only a few studies describe this subtype in a small number of CRC patients [21–24]. EMAST has not been linked to major defects in DNA mismatch repair. Heterogeneous and reduced protein expression of MSH3 was observed in association with EMAST in CRC [21–24]. More recent reports suggest that MSH3 deficiency is the cause of EMAST in human CRC cells [25,26]. The link between MSH3 and EMAST suggests an acquired effect, as no germ line mutation in *MSH3* has ever been demonstrated [4]. There is a broad range in the prevalence of EMAST is CRC and the biological significance of EMAST in CRC is not clear. Only one article described an association with outcome for stage II/III CRC patients.[27]

Only limited data is available regarding EMAST or MSH3 expression in CRC patients. In the current study we evaluated the frequency of EMAST in MSI and microsatellite stable (MSS) CRC tumors. In addition, we assessed in an exploratory analysis the role of EMAST as a prognostic biomarker in metastatic CRC (mCRC) patients.

## Material and Methods

### Patient populations

**Fig 1:**
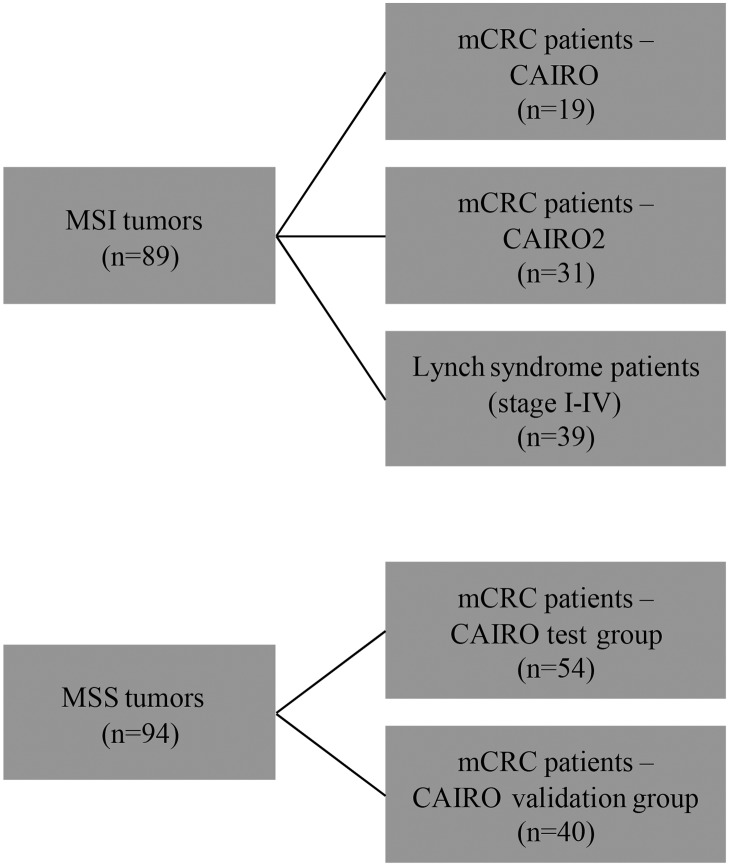
Flowchart of selected CRC patients to determine the frequency and prognostic value of EMAST.

Data were derived from mCRC patients included in two large phase III studies: CAIRO (ClinicalTrials.gov NCT00312000) (n = 820) and CAIRO2 (n = 755) (ClinicalTrials.gov NCT00208546), of which the results have been published previously [28,29]. Collection of formalin-fixed paraffin-embedded (FFPE) material of the primary tumor was part of the initial protocol in both studies. To determine the frequency and prognostic value of EMAST in mCRC patients with MSI tumors we selected 50 mCRC patients with MSI tumors treated in both CAIRO studies. Since MSI is relatively rare in mCRC we combined the patients of the CAIRO (n = 19) and the CAIRO2 (n = 31) study. No validation cohort could be selected for MSI patients. To further evaluate the relation between EMAST and MSI, we retrieved 39 tumors from CRC patients (anonymous samples) with known Lynch syndrome (stage I-IV) from our own database (that has been set up conform the guidelines of the local medical ethical committee (Commissie Mensgebonden Onderzoek Radboudumc) with written informed consent of the patients, from which use of tissue is approved for this study). To determine the frequency and prognostic value of EMAST in mCRC patients with MSS tumors we selected 54 patients of the CAIRO study with comparable characteristics (test group). Patients within the test group were all treated with first-line capecitabine monotherapy for at least 3 cycles, localization of the primary tumor in colon or recto- sigmoid which was resected, WHO performance score 0, normal baseline serum lactate dehydrogenase (LDH) concentration, and had not received prior adjuvant chemotherapy. In addition, we randomly selected 40 additional mCRC patients with MSS tumors treated in the same CAIRO study as a validation group. (Fig 1)

### EMAST analysis

Genomic DNA was extracted from four to eight manually microdissected 30 μm section of FFPE tissue of the primary tumors. Areas containing >50% tumor cells were selected by microscopic evaluation on a reference slide stained with H&E. Genomic DNA from microdissected tissues was isolated using the QIAamp DNA micro kit (Qiagen, Valencia, CA) following the manufacturer’s instructions. DNA concentration was determined at 260 nm using the Nanodrop ND-1000 spectrophotometer (Nanodrop Technologies, Inc., Wilmington, DE, USA). EMAST analysis was performed in duplicate on normal and tumor DNA of the selected patients. EMAST status was determined by PCR and GeneScan analysis using five tetranucleotide markers: MYCL1, D8S321, D9S242, D20S82 and D20S85 (S1 Table) [23]. A tumor was defined EMAST if at least two of the five markers showed instability and non-EMAST if only one or none of the markers showed instability [22].

Patients were analyzed for the frequency and prognostic value in four different groups: patients with combined MSI and EMAST tumors (MSI / EMAST), patients with combined MSI and non-EMAST tumors (MSI / non-EMAST), patients with combined MSS and EMAST tumors (MSS / EMAST) and patients with combined MSS and non-EMAST tumors (MSS / non-EMAST). The frequency of EMAST was compared for patients with MSI and MSS tumors. The outcome was analyzed within the group of patients with MSI tumors (excluding the Lynch syndrome patients) for EMAST compared to non-EMAST tumors and within the group of patients with MSS tumors for EMAST compared to non-EMAST tumors.

### Immunohistochemistry MSH3

Immunohistochemistry (IHC) was performed on tissue microarrays (TMA) of the primary tumors of 549 eligible randomized patients in the CAIRO study as previously described.[30] 4 μm slides were cut of every TMA and mounted on glass. Xylene and ethanol were used for deparaffinization and dehydration of the TMA slides. Water and phosphate-buffered saline (PBS) were used for washing of the slides. Endogenous peroxidase activity was blocked with 3% hydrogen peroxide in PBS for 30 min and slides were washed with water, after which heat-induced epitope retrieval was performed. The slides were stained with a monoclonal antibody against MSH3 (clone ERP4334; Epitomics—an Abcam company, Burlingame, CA, USA), dilution 1:5000. Two independent investigators performed the scoring, and if the slide scoring was not unambiguous, the opinion of a third investigator (pathologist IDN) was final. Staining pattern of the MSH3 protein was evaluated by using the normal epithelial, stromal and inflammatory cells as internal control. Low MSH3 protein expression was defined as <85% brown staining of cell cores in tumor cells and high MSH3 protein expression was defined as ≥85% brown staining of cell cores in tumor cells and not applicable if neither tumor nor stromal cells showed MSH3 protein expression [21].

### MSI, hypermethylation of the MLH1 gene promoter and BRAF status

For samples of both CAIRO studies, immunohistochemistry (IHC) was performed on FFPE tissue with antibodies against MLH1, MSH2, MSH6 and PMS2. In addition, MSI analysis was performed where there was an absence of MMR protein expression or equivocal IHC results. MSI status was determined using two microsatellite markers (BAT 25 and BAT 26). If only one of these markers showed instability, the analysis was extended with four additional markers (BAT 40, D2S123, D5S346, and D17S250). A tumor was defined as MSI if at least two of the six markers showed instability or MSS if none of the markers showed instability. Tumors with only one of the markers showing instability were defined as MSI-low and included in the MSS category.[30] Hypermethylation status of the *MLH1* gene promoter and the *BRAF* V600E mutation status, was assessed as described previously [30–32].

### Statistical analysis

For the EMAST analysis, patients were divided into two categories: EMAST and non-EMAST tumors. The association between EMAST and MSH3 protein expression was investigated with a logistic regression model with independent factors group and MSH3 expression. OS was defined as the time from the date of randomization to the date of death from any cause. OS curves were estimated using the Kaplan—Meier method and compared using a Cox proportional hazard model. All tests were two-sided and p<0.05 was considered as statistically significant. All analyses were conducted using the SAS system version 9.2.

### Literature search strategy, inclusion criteria, and data extraction

We reviewed the literature on the frequency of EMAST in CRC patients with MSI and MSS tumors. A search was conducted of Medline, PubMed, and the Cochrane Library from January 1990 to April 2014 with an English-language restriction, using the following search terms: EMAST, tetranucleotide repeat, in combination with colon cancer and colorectal cancer. Original publications were selected if the abstract contained data for patients with EMAST. In case of duplicate publications, the most recent and/or most complete study was included. Publications were excluded if frequency of EMAST was limited to either patients with MSI or MSS tumors.

## Results

### Prevalence of EMAST

Overall, EMAST was observed among 45.9% of a total of 183 tumors (Table 1). Frequency of EMAST was significantly higher among patients with MSI tumors compared to MSS tumors: 79.8% compared to 13.8% (p<0.001).

**Table 1:** Prevalence of EMAST and non-EMAST tumors in the different patient groups.

		EMAST	non-EMAST	Total number of patients	*p* value
	**Patients with MSI tumors**				
	mCRC	84.0%	16.0%	50	
	Lynch syndrome	74.4%	25.6%	39	0.113
	Total	79.8%	20.2%	89	
	**Patients with MSS tumors**				
	Test group of patients	11.1%	88.9%	54	
	Validation group of patients	17.5%	82.5%	40	0.160
	Total	13.8%	86.2%	94	
All patients	Total	45.9%	54.1%	183	

*p* value represent heterogeneity between groups

Abbreviations: EMAST = elevated microsatellite alterations at selected tetranucleotide repeats, MSI = microsatellite instability, MSS = microsatellite stability

**Fig 2:**
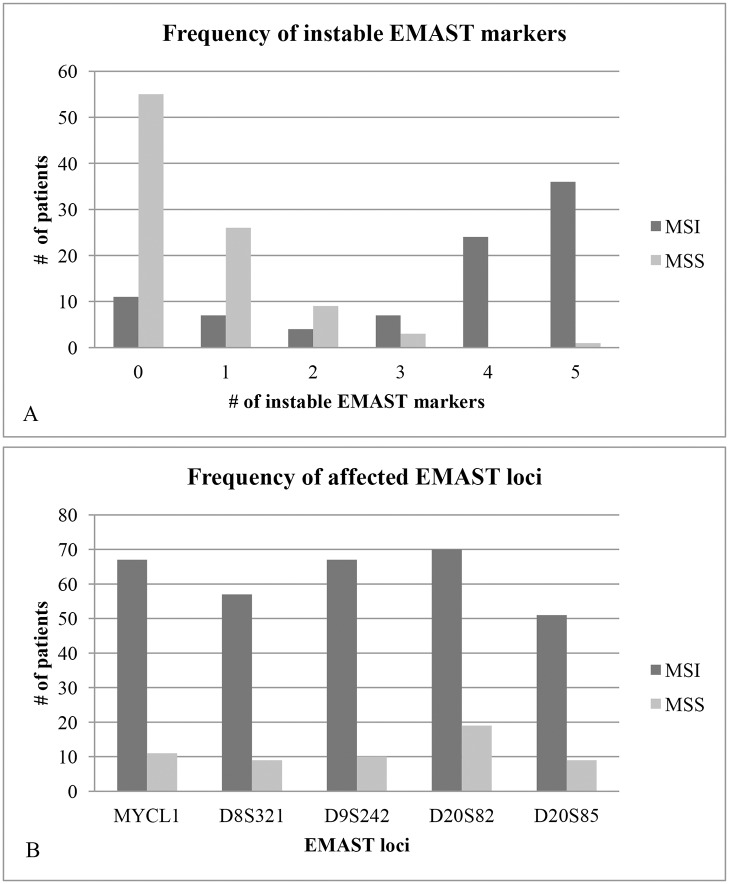
Frequency of instable EMAST markers (A) and frequency of affected EMAST loci (B), subdivided by patients with MSI and MSS tumors.

In patients with MSS / EMAST tumors instability was generally shown at 2 EMAST loci (69.2%, 9 out of 13), whereas in patients with MSI / EMAST tumors instability was frequently shown at 4 (33.8%, 24 out of 71), or 5 (50.7%, 36 out of 71) EMAST loci (Fig 2A). The highest frequency of instability in EMAST tumors was demonstrated at the D20S82 locus (91.7%, 77 out of 84), followed by the MYCL1 locus (86.9%, 73 out of 84), the D9S242 locus (84.5%, 71 out of 84), the D8S321 locus (72.6%, 61 out of 84) and the D20S85 locus (65.5%, 55 out of 84) (Fig 2B).

### EMAST and MSH3

**Fig 3:**
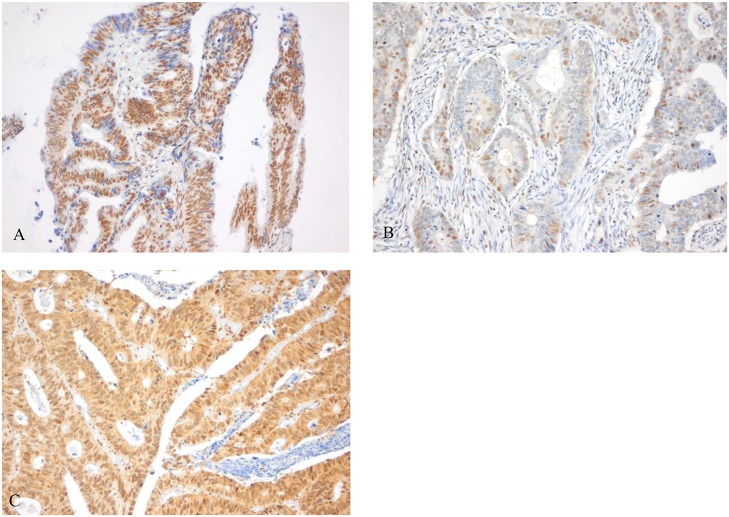
Staining pattern of MSH3 protein expression. Heterogeneous MSH3 protein expression (A), demonstrated by expression of both brown (positive) and blue (negative) nuclei upon MSH3 IHC staining. Low MSH3 protein expression was defined as <85% brown staining of cell cores in tumor cells (B) and high MSH3 protein expression was defined as ≥85% brown staining of cell cores in tumor cells (C).

The majority of mCRC patients (n = 381, 69.4%) had a high expression of MSH3 in tumor cells (Fig 3). 21.1% of tumors demonstrated nuclear heterogeneity by expression of both positive and negative nuclei upon MSH3 IHC staining (Fig 3A–3C). Both MSH3 expression and EMAST status was known in 139 patients. Heterogeneous or high MSH3 protein expression was not correlated to EMAST status (p = 0.088 and p = 0.856, respectively).

### Outcome of patients with MSI tumors

Patients with MSI / EMAST tumors were mostly female (52% versus 22%, respectively, p = 0.038) (Table 2). Moreover, EMAST tumors were more frequently located above the rectosigmoid area (93% versus 63%, p = 0.006).

**Table 2:** Baseline patient and tumor characteristics of patients with MSI and MSS tumors, subdivide by EMAST and non-EMAST tumors.

	Patients with MSI tumors			Patients with MSS tumors					
				Test group			Validation group		
	EMAST	non-EMAST		EMAST	non-EMAST		EMAST	non-EMAST	
	n = 42	n = 8	*p* value	n = 6	n = 48	*p* value	n = 7	n = 33	*p* value
**Median age (range)**	68 (34–84)	59 (37–73)	0.131	72 (47–77)	66 (34–79)	0.749	71 (58–76)	67 (39–81)	0.161
**Sex**									
male	20 (48%)	7 (88%)	**0.038**	5 (83%)	31 (65%)	0.651	5 (71%)	22 (67%)	0.338
female	22 (52%)	1 (22%)		1 (17%)	17 (35%)		2 (29%)	11 (33%)	
**WHO performance status**									
PS0	26 (62%)	4 (50%)	0.156	6 (100%)	48 (100%)	-	3 (42%)	22 (67%)	**0.045**
PS1	14 (33%)	4 (50%)		-	-		3 (42%)	11 (33%)	
PS2	2 (5%)	-		-	-		1 (16%)	-	
**Serum LDH**									
normal	32 (76%)	4 (50%)	0.110	6 (100%)	48 (100%)	-	5 (71%)	23 (70%)	0.348
abnormal	10 (24%)	4 (50%)		-	-		2 (29%)	10 (30%)	
**Previous adjuvant therapy**									
yes	5 (12%)	2 (25%)	0.239	-	-	-	3 (42%)	5 (15%)	0.108
no	37 (88%)	6 (75%)		6 (100%)	48 (100%)		4 (58%)	28 (85%)	
**Localization of the primary tumor**									
colon	93 (93%)	5 (63%)	**0.006**	5 (83%)	42 (88%)	0.416	3 (42%)	20 (61%)	0.869
recto sigmoid	-	2 (25%)		1 (17%)	6 (12%)		-	1 (3%)	
rectum	2 (5%)	1 (12%)		-	-		4 (58%)	11 (33%)	
multiple tumor				-	-		-	1 (3%)	
unknown	1 (2%)	-							
**Histology of the primary tumor**									
adenocarcinoma	22 (52%)	5 (63%)	0.144	5 (83%)	35 (73%)	0.183	7 (100%)	24 (73%)	0.141
mucinous adenocarcinoma (>50% WHO)	16 (38%)	2 (25%)		1 (17%)	4 (8%)		-	4 (12%)	
adenocarcinoma + mucinous component	4 (10%)	-		-	6 (13%)		-	4 (12%)	
other	-	1 (12%)		-	3 (6%)		-	1 (3%)	
***BRAF* mutation status**									
mutation	24 (57%)	-	**0.004**	-	5 (11%)	0.590	-	-	0.677
wild-type	16 (38%)	7 (88%)		6 (100%)	40 (83%)		7 (100%)	31 (94%)	
unknown	2 (5%)	1 (12%)		-	3 (6%)		-	2 (6%)	

NOTE: Statistically significant results are set in bold

Abbreviations: MSI = microsatellite instability, MSS = microsatellite stability, EMAST = elevated microsatellite instability at selected tetranucleotide repeats

Median OS was significantly worse for patients with MSI / EMAST compared to MSI / non-EMAST tumors treated in both CAIRO studies (11.4 versus 39.4 months, respectively, HR 3.22, 95% CI 1.25–8.30) (Table 3).

**Table 3:** Overall survival of patients with MSI and MSS tumors, subdivided by EMAST and non-EMAST tumors.

	EMAST	non-EMAST	HR	
	(months (95% CI))	(months (95% CI))	(95% CI)	*p* value
MSI tumors	n = 42	n = 8		
	11.4 (7.3–15.6)	39.4 (8.0-NE)	3.22 (1.25–8.30)	**0.010**
Test group (MSS tumors)	n = 6	n = 48		
	22.4 (22.4-NE)	19.1 (14.4–23.7)	0.33 (0.10–1.07)	0.053
Validation group (MSS tumors)	n = 7	n = 33		
	19.3 (8.4–38.6)	18.2 (14.6–21.9)	1.14 (0.50–2.61)	0.759

NOTE: Statistically significant results are set in bold

Abbreviations: EMAST = elevated microsatellite instability at selected tetranucleotide repeats, MSI = microsatellite instability, MSS = microsatellite stability, CI = confidence interval, HR = hazard ratio, NE = not estimable

### MSI / EMAST and the relation to MMR proteins

**Fig 4:**
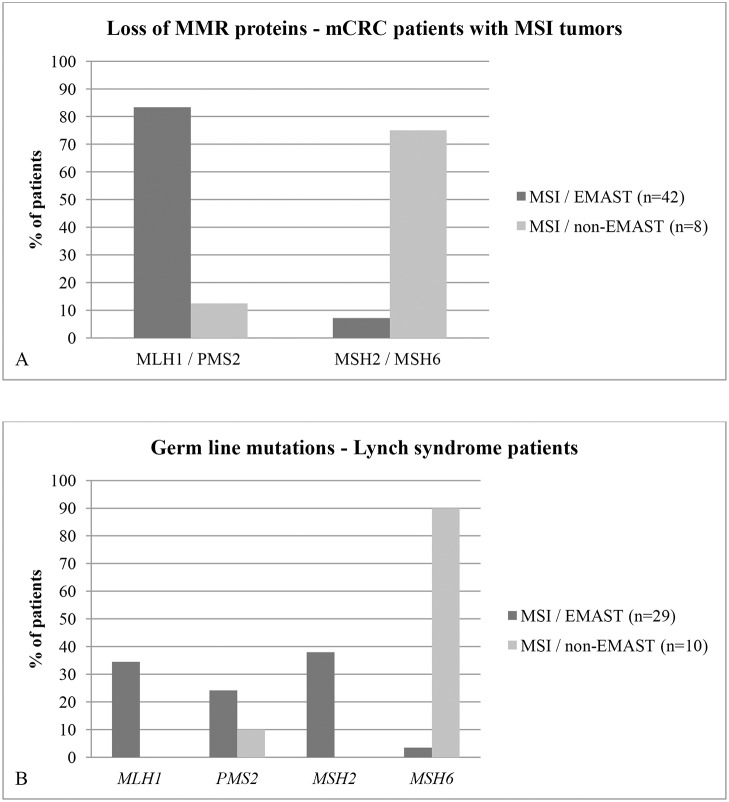
Percentage of mCRC patients with MSI tumors and loss of MLH1 and/or PMS2 (MLH1 / PMS2) and MSH2 and/or MSH6, (MSH2 and MSH6) subdivided in patients with MSI / EMAST tumors and patients with MSI / non-EMAST tumors. (A). Percentage of patients with known Lynch syndrome and germ line mutation of the different MSI genes, subdivided in patients with MSI / EMAST tumors and patients with MSI / non-EMAST tumors (B).

The distribution of loss of MMR proteins in mCRC tumors is summarized in Fig 4A. Most patients with MSI tumors showed loss of MLH1 and/or PMS2 protein expression (72.0%, 36 out of 50 patients). Loss of MSH2 and/or MSH6 protein expression was found in 18.0% (9 out of 50) of patients. These patients are likely Lynch or Lynch-like syndrome patients. Only 7.1% (3 out of 42) of patients with a MSI / EMAST tumor showed loss of expression of the MSH6 protein, compared to 62.5% (5 out of 8) of patients with MSI / non-EMAST tumors.

Hypermethylation of the *MLH1* gene promoter (32 out of 40 patients) and *BRAF* mutations (24 out of 40 patients) were limited to the patients with a MSI / EMAST tumor (p < 0.001 and p = 0.004 respectively).

### EMAST in patients with Lynch syndrome

In order to further analyze the relation of MSI and EMAST we selected 39 patients with known Lynch syndrome (with germ line mutations in *MSH2* gene (n = 11), *MLH1* gene (n = 10), *MSH6* gene (n = 10) and *PMS2* gene (n = 8)). The majority of this population showed EMAST (29 out of 39 patients). None of the patients showed hypermethylation of the *MLH1* gene promoter, and all tumors were *BRAF* wild-type. Nine out of 10 patients presenting with non-EMAST tumors had a germline mutation in *MSH6* (Fig 4B).

### Outcome of patients with MSS tumors

Baseline patient and tumor characteristics for patients with MSS tumors (test group and validation group), subdivided by EMAST and non-EMAST tumors are presented in Table 2. There was no significant difference in outcome for patients with EMAST compared to patients with non-EMAST tumors (Table 3).

### Review of the literature

**Fig 5:**
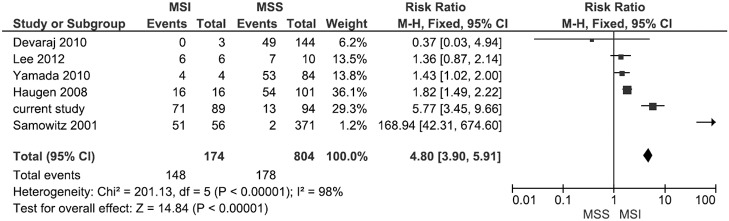
Forest plot for the association of prevalence of EMAST in patients with MSI compared to MSS tumors in stage I-IV CRC.

The literature search identified 7 studies in which EMAST was described in stage I-IV CRC patients with MSI and MSS tumors [21–24,27,33,34]. Two studies were excluded: one study described the same population [23] and one study assessed the prevalence of EMAST solely in patients with MSS tumors [27]. Three studies had limited numbers of MSI tumors. Fig 5 summarizes a forest plot of the 5 published studies and the current study on the prevalence of EMAST in stage I-IV CRC patients with MSI and MSS tumors. EMAST is significantly more frequent in tumors with MSI (148/174) (RR 4.80, 95% confidence interval 3.90–5.91). Significant heterogeneity was observed.

## Discussion

This study presents the analysis on the frequency and prognostic value of EMAST in mCRC patients. Although EMAST was observed in 45.9% of all mCRC patients, it was most pronounced in MSI tumors (79.8%). The frequency of EMAST among MSS tumors (13.8%) was much lower in our study compared to most studies in stage I-IV CRC [21–24,27,34]. The broad range of frequency (0.54–60.2%) of EMAST among MSS tumors described in literature [21–24,27,33,34] might be due the fact that there is no consensus on the definition of EMAST and the panel required for its diagnosis. Because of the polymorphic nature of tetranucleotide repeats in the current study we used stringent criteria for the definition of EMAST: at least two of the five tetranucleotide markers should show instability.

Despite the fact that several small studies (n = 3 to n = 56) [21–24,33,34] demonstrated that MSI invariably is associated with EMAST, the correlation between EMAST and MSI is not widely accepted. We found a high frequency of EMAST in MSI tumors, which was confirmed in a analysis of the existing literature in stage I-IV CRC (Fig 5). Only a small subset of patients with MSI tumors is non-EMAST. Interestingly, the majority of patients with MSI / non-EMAST tumors showed loss of MSH6. This is in line with the fact that MSH6 is only involved in mononucleotide mismatch repair [35,36]. Patients with MSI / non-EMAST tumors are more often male and tumors developed more frequently in the rectum, these characteristics are comparable to patients with *MSH6* germline mutations [37]. In our population of MSI tumors the presence of EMAST is correlated with *MLH1* deficiency, which causes a total DNA mismatch repair defect, both in Lynch syndrome as well as in the sporadic setting, confirming earlier observations [21,27].

Data about the EMAST phenotype in CRC are scarce and underlying mechanism(s) remain unclear. The earliest reports suggested that EMAST might be associated with mutations in the *TP53* gene [16,17] or that environmental carcinogens may exacerbate this phenotype [38] in cancers other than CRC. Later on, an association was made between loss of MSH3 and EMAST in CRC [21]. Due to the fact that MutSβ has a strong affinity for recognizing more than two unpaired nucleotides and genetic complementation of MSH3 deficiency in human cells increased stability at loci containing dinucleotide and tetranucleotide repeats [39], it was argued that that loss of MutSβ due to MSH3 inactivation may result in MSI not only at loci containing dinucleotide repeats, but also at loci with tetranucleotide repeats, such as EMAST [21]. Recent studies confirm the association between loss of MSH3 and EMAST in CRC, however the exact underlying mechanism remains unknown [25,26]. We failed to demonstrate a correlation between MSH3 protein expression and EMAST. Actually, we did find the heterogeneous expression pattern of MSH3 in 21.1% of patients as described by others, although this was not correlated to EMAST. However our study had a small number of patients. Another possible explanation for the difference between our study and previous studies might be a potential undersampeling bias since we used one TMA specimen per tumor instead of full tumor slides.

We demonstrate that patients with MSI / non-EMAST tumors had a significantly better prognosis compared to patients with MSI / EMAST tumors. The addition of EMAST to MSI tumors seems to worsen overall prognosis and survival. The MSI / non-EMAST group would be expected to be enriched for *MSH6* deficiency since EMAST would be identified with a total DNA mismatch repair defect, such as with *MLH1* or *MSH2* deficiency. Our results on the correlation of EMAST with clinical outcome should be interpreted with caution due to the small number of patients in the different subgroups.

In summary, the frequency of EMAST among mCRC patients with MSS tumors is low compared to patients with MSI tumors. There was no correlation between EMAST and MSH3 protein expression. We did find a clear link between MSI and EMAST, and outcome was significantly better for mCRC patients with MSI / non-EMAST tumors. Further studies are warranted to elucidate the molecular basis for EMAST and to show whether the tetranucleotide alterations observed in EMAST tumors may have a functional impact by themselves or just represent bystander alterations in a subset of tumors with specific defects in DNA replication and repair.

## Supporting Information

S1 Table Tetranucleotide microsatellite PCR primer sequences. (PDF)
