# Supplementary material for: EMAST Is Associated with a Poor Prognosis in Microsatellite Instable Metastatic Colorectal Cancer
Source: PLoS One. 2015 Apr 17;10(4):e0124538. doi: 10.1371/journal.pone.0124538 (PMC4401564; doi:10.1371/journal.pone.0124538)
Supplement: S1 Table — (PDF) [file pone.0124538.s001.pdf]

Primers	Sequence	Annealing temperature	Product size (bp)
MYCL1	Fwd: TGG CGA GAC TCC ATC AAA G	56.0	181
	Rev: CCT TTT AAG CTG CAA CAA TTT C	56.0	181
D20S82	Fwd: GCC TTG ATC ACA CCA CTA CA	59.0	249
	Rev: GTG GTC ACT AAA GTT TCT GCT	59.0	249
D20S85	Fwd: GAG TAT CCA GAG AGC TAT TA	56.0	146
	Rev: ATT ACA GTG TGA GAC CCT G	56.0	146
D8S321	Fwd: GAT GAA AGA ATG ATA GAT TAC AG	53.0	237
	Rev: ATC TTC TCA TGC CAT ATC TGC	53.0	237
D9S242	Fwd: GTG AGA GTT CCT TCT GGC	56.0	178
	Rev: ACT CCA GTA CAA GAC TCT G	56.0	178
